# Assessing fluid responsiveness with ultrasound in the neonatal intensive care setting: the mini-fluid challenge

**DOI:** 10.1007/s00431-024-05425-6

**Published:** 2024-01-26

**Authors:** Koert de Waal, Enrico Petoello

**Affiliations:** 1 Department of Neonatology, John Hunter Children’s Hospital, University of Newcastle, Lookout road, New Lambton, Newcastle, NSW 3205 Australia; 2 Neonatal Intensive Care Unit, Azienda Ospedaliera Universitaria Integrata Verona, Verona, Italy

**Keywords:** Sepsis, Newborn, Fluid challenge, Ultrasound

## Abstract

The mini-fluid challenge (MFC) can guide individualised fluid therapy and prevent fluid overload and associated morbidity in adult intensive care patients. This ultrasound test is based on the Frank-Starling principles to assess dynamic fluid responsiveness, but limited MFC data exists for newborns. This brief report describes the feasibility of the MFC in 12 preterm infants with late onset sepsis and 5 newborns with other pathophysiology. Apical views were used to determine the changes in left ventricular stroke volume before and after a 3 ml/kg fluid bolus was given over 5 min. Four out of the 17 infants were fluid responsive, defined as a post-bolus increase in stroke volume of 15% or more.

*Conclusion*: The MFC was feasible and followed the physiological principles of stroke volume and extravascular lung water changes and 24% were fluid responsive. The MFC could enable future studies to examine whether adding fluid responsiveness to guide fluid therapy in newborns can reduce the risk of fluid overload.

**Table:** 

**What is Known:** *• Fluid overload is associated with morbidity and mortality.* *• The mini-fluid challenge (MFC) provides a personalised approach to fluid therapy.*	
**What is New:** *• The MFC is feasible in newborns.* *• The MFC followed the physiological principles of stroke volume and extravascular lung water changes.*

**Supplementary Information:**

The online version contains supplementary material available at 10.1007/s00431-024-05425-6.

## Background

There are various clinical reasons why we prescribe a fluid bolus in the neonatal intensive care setting, e.g. shock, hypotension, poor perfusion or metabolic acidosis [1]. However, our current approach to fluid therapy is associated with a high risk of fluid overload and related adverse clinical outcomes, especially in newborn infants with sepsis or post-surgery [2, 3]. Volume overload is associated with increased mortality and increased need for respiratory support; hence, there is a pressing need for a more individualised approach to fluid therapy [4].

The two main pathophysiological reasons to prescribe a fluid bolus are (1) to restore low intravascular volume and (2) to increase preload with the aim to increase stroke volume. Intravascular volume status can be difficult to estimate. The venous system can be divided into a stressed volume compartment that contributes to generating central venous pressure and preload and an unstressed volume compartment that does not generate pressure but can be recruited in times of need. As true hypovolemia with low intravascular volume is rather uncommon in neonatology, most clinical scenarios where the clinician decides to prescribe a fluid bolus are in patients with relative hypovolemia with vasodilatation, the predominant pathophysiology found in sepsis or post-surgical inflammation. Much of the intravascular volume still resides within the venous system, but it is not always clear how the intravascular volume is distributed between the stressed and unstressed compartments and whether fluid therapy can restore central venous pressure and preload [5].

Fluid responsiveness, i.e. stroke volume changes with alternations in preload conditions, is difficult to determine with clinical examination alone. There is a growing amount of evidence that supports the use of bedside-focused ultrasound to estimate whether a fluid bolus would end up as stressed or unstressed volume and thus determine fluid responsiveness [6]. One method of measuring fluid responsiveness with ultrasound is by giving a rapid small fluid bolus and measuring the change in stroke volume immediately after. This so called mini-fluid challenge (MFC) uses the Frank-Starling principles whereby at lower preload before a fluid bolus is given, a proportionally greater increase in stroke volume is expected after the fluid bolus. The aim of this pilot study is to test the feasibility of a standardised mini-fluid challenge in the neonatal intensive care setting.

## Methods

Any newborn who was prescribed a fluid bolus for clinical reasons was included in this pilot study that ran from August 2022 to June 2023 in the neonatal department of the John Hunter Children’s Hospital. Ethical approval for this study was obtained from the Hunter New England human research ethics committee (2022/STE03027).

**Fig. 1:**
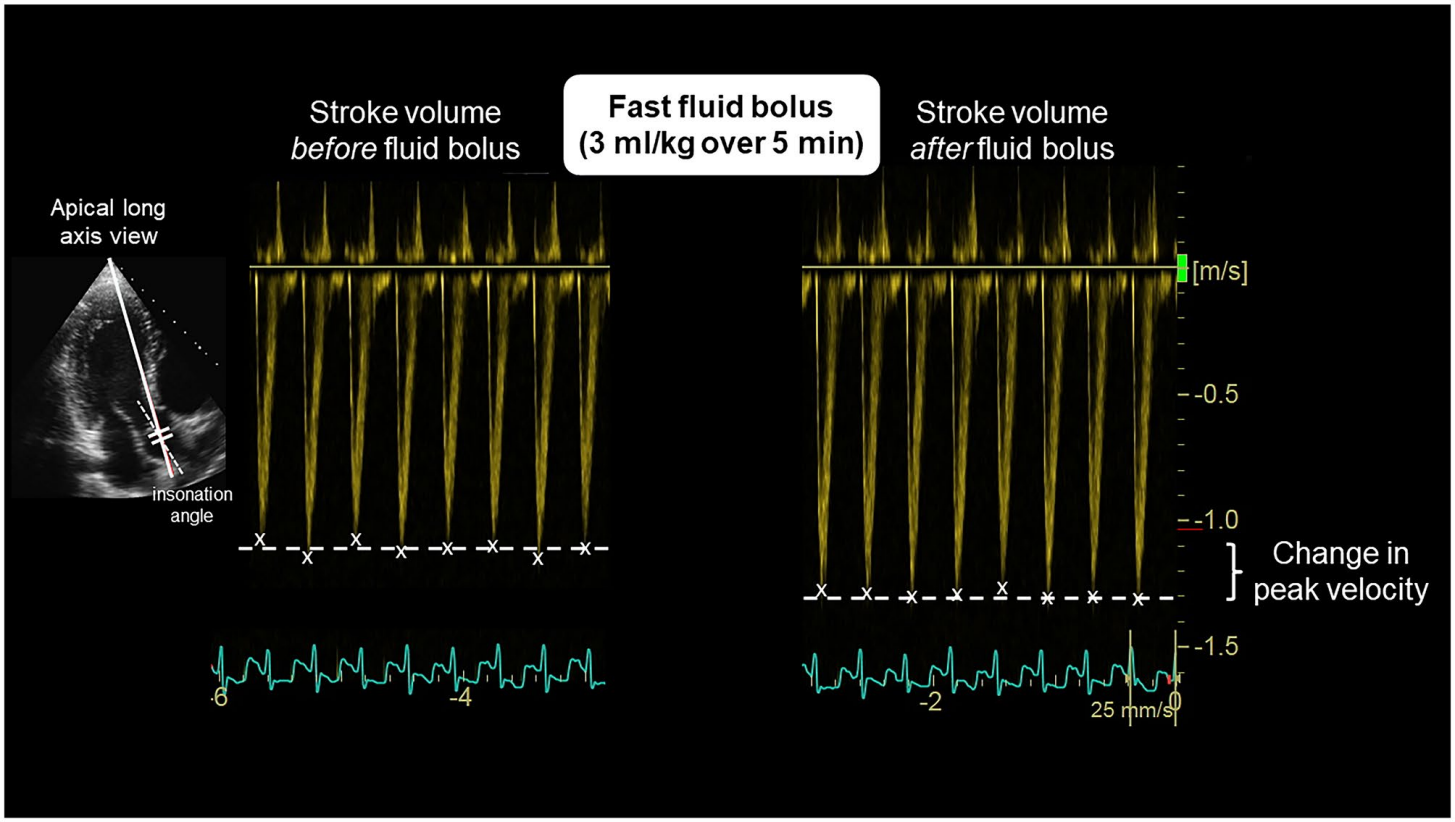
Example of a mini-fluid challenge (MFC) in a preterm infant. The apical long axis was used to visualise the aorta outflow tract. Pulse wave Doppler with minimal angle of insonation was added to capture stroke volume and determine the maximum velocity from 10 to 15 cardiac cycles at low sweep speed

The MFC was achieved with 3 ml/kg of normal saline given over 5 min. The apical long axis was used to visualise the aorta outflow tract. Pulse wave Doppler with minimal obtainable angle of insonation was added to capture stroke volume and determine the average maximum velocity from 10 to 15 cardiac cycles at low sweep speed (Fig. 1). Particular effort was made to maintain the same angle of insonation on the before and after ultrasound, and images with more than 10° difference in angle of insonation were excluded from analysis [7]. The definition of fluid responsiveness was set at more than 15% increase in stroke volume as per MFC recommendations in children and adults [8, 9].

The findings of the MFC were not shared with the clinical team and not used to guide further therapies. Data on further fluid administration, change in cardiovascular medications and the oxygenation saturation–derived oxygenation index (OSI, = 2 × mean airway pressure × FiO_2_ / saturation) as proxy for respiratory requirement was collected 4 h after the MFC. Analysis was performed with paired sample *t* test on GraphPad version 6 (Prism, LaJolla, CA, USA).

## Results

**Fig. 2:**
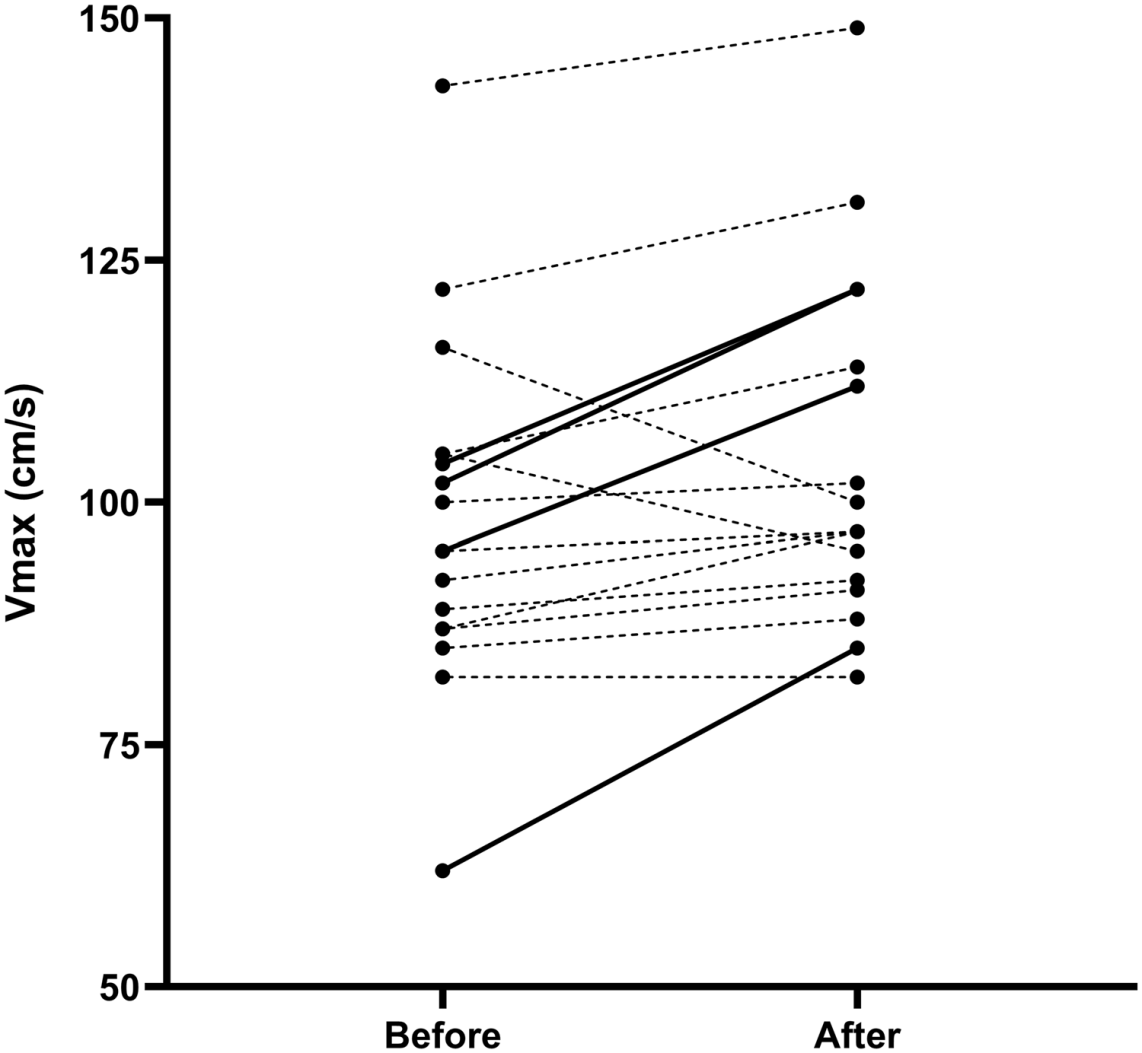
Average maximum doppler velocity in the aorta outflow tract before and after the mini-fluid challenge. Solid lines indicate a rise > 15% and thus fluid responsiveness

Twelve preterm infants with late onset sepsis and 5 infants with other pathophysiology were included in this pilot data. The patient demographics and characteristics before the MFC are presented in supplemental Table 1 and 2. All infants with late onset sepsis increased stroke volume with the MFC, and 4 out of 17 infants could be classified as fluid responders as per our definition (Fig. 2). The change in oxygenation index in the 4 h after the MFC was higher in the infants with sepsis who were non-responders when compared to the responders (OSI + 0.2 versus + 0.9, *p* < 0.02) suggesting a possible increase in extravascular lung water. Fluid responsiveness was not associated with clinical outcomes such as further fluid therapy or the initiation of cardiovascular medications.

## Discussion

Our pilot data showed that the mini-fluid challenge is feasible in the neonatal intensive care setting and that 23% were fluid responsive. The MFC can be applied by any clinician after a brief period of training in focused point-of-care ultrasound, but extra attention is required to maintain the same angle of insonation in the before and after scan.

In preterm infants with late onset sepsis, a disease characterised by vasodilatation and relative hypovolemia, our findings suggest that the MFC followed the physiological principles of stroke volume and extravascular lung water changes. More data is needed to determine whether the MFC could help predict if further fluid boluses are likely to end up as stroke volume (responders) or contribute to an increased need for respiratory support (non-responders). Alternative measurement tools are available to explore fluid therapy such as electrical cardiometry for stroke volume changes and lung ultrasound for accumulation of extravascular lung water and could be added in future studies [10, 11].

The MFC has been extensively studied in adult intensive care patients [9, 12, 13]. Various alternative methods to test fluid responsiveness are available, e.g. by changing preload conditions with mechanical ventilation or with the passive leg raising test. The MFC was proven to be the most accurate when compared to invasive measurements, and when given as a small bolus in less than 4 min [14]. In children and in the only available study in newborns, the fluid bolus studied was generally large (10–20 ml/kg) and given over 30–60 min, and thus not reaching the definition of a MFC and might not provide the same level of accuracy as a diagnostic test [8].

Several details of the MFC need further study in newborns to optimise this diagnostic test. The volume bolus, rapid infusion time and definition of fluid responsiveness were derived from supportive evidence gathered in adults. However, newborns have rather compliant vessels with high cardiac output, and thus, an altered cut point of what constitutes fluid responsiveness might be required. The amount of fluid and timing (3 ml/kg over 5 min) is comparable to other medications in the neonatal intensive care (e.g. gentamicin), but the safety of a fast fluid bolus could be a concern in very preterm infants with compromised cerebral autoregulation early after birth.

Our pilot study is too small to make meaningful comments about clinical outcomes in relation to fluid responsiveness, and we have started an international multicentre study to explore further research questions. We hypothesise that the MFC enhances the clinicians’ ability to determine the need for fluid compared to clinical parameters alone. When fluid responsiveness was added as systematic assessment in adult patients with septic shock, further fluid boluses could be avoided in non-fluid responsive patients without any negative impact on clinically relevant outcomes [15, 16]. Future studies will have to determine whether adding the MFC to test fluid responsiveness and guide treatments can reduce the risk of fluid overload in newborns as well.

### Supplementary Information

Below is the link to the electronic supplementary material.

Supplementary file1 (DOCX 25 KB)

## Data Availability

The data that supports the findings of this study are available from the corresponding author upon reasonable request.
