# Supplementary material for: Assessing fluid responsiveness with ultrasound in the neonatal intensive care setting: the mini-fluid challenge
Source: Eur J Pediatr. 2024 Jan 26;183(4):1947–51. doi: 10.1007/s00431-024-05425-6 (PMC11001719; doi:10.1007/s00431-024-05425-6)
Supplement: Supplementary file 1 — Supplementary file1 (DOCX 25 KB) [file 431_2024_5425_MOESM1_ESM.docx]

| Patient | GA (weeks) | Birth weight (gram) | Sex | Antenatal steroids | Mode of birth | 5 min Apgar | Postnatal age (days) | PDA (mm) | IVH grade | NEC | BPD grade |
| --- | --- | --- | --- | --- | --- | --- | --- | --- | --- | --- | --- |
| 1 | 24 | 665 | M | 2 | vaginal | 5 | 17 | 1.9 | 1 | 0 | 2 |
| 2 | 25 | 850 | M | 1 | CS | 8 | 18 | 2.0 | 0 | 1 | 1 |
| 3 | 25 | 945 | M | 2 | CS | 8 | 8 | 2.1 | 0 |  |  |
| 4 | 26 | 570 | M | 2 | CS | 6 | 7 | 0.0 | 0 | 1 | 2 |
| 5 | 27 | 650 | F | 2 | CS | 7 | 5 | 1.8 | 0 | 0 | 2 |
| 6 | 25 | 810 | F | 1 | CS | 6 | 18 | 2.0 | 0 | 0 | 1 |
| 7 | 28 | 1260 | M | 2 | vaginal | 9 | 3 | 0.0 | 0 | 0 | 0 |
| 8 | 28 | 1260 | M | 2 | vaginal | 9 | 4 | 0.0 | 0 | 0 | 0 |
| 9 | 25 | 860 | M | 2 | CS | 9 | 8 | 0.8 | 0 | 0 | 1 |
| 10 | 26 | 540 | F | 2 | CS | 7 | 6 | 2.0 | 1 | 0 | 2 |
| 11 | 27 | 1030 | M | 2 | CS | 9 | 20 | 0.0 | 2 | 0 | 2 |
| 12 | 25 | 630 | F | 1 | CS | 6 | 10 | 1.0 | 3 | 0 | 1 |
|  |  |  |  |  |  |  |  |  |  |  |  |
| 13 | 34 | 2210 | F | 1 | CS | 9 | 2 | 0.5 |  |  |  |
| 14 | 37 | 2180 | M | 0 | CS | 9 | 2 | 0.0 |  |  |  |
| 15 | 32 | 1625 | F | 1 | CS | 6 | 5 | 2.5 |  |  |  |
| 16 | 41 | 4512 | M | 0 | vaginal | 5 | 1 | 0.0 |  |  |  |
| 17 | 36 | 2700 | M | 0 | CS | 0 | 1 | 0.0 |  |  |  |

Table 1. (Supplemental material) Patient demographics. GA, gestational age; PDA, patent ductus arteriosus; IVH, intraventricular haemorrhage; NEC, necrotising enterocolitis; BPD, bronchopulmonary dysplasia. CS, caesarean section.

| Patient | Clinical | Indication | Blood culture | Bolus number | iv access route used | HR  (/min) | SBP (mmHg) | DBP (mmHg) | pH | Lactate (mmol/l) | OSI |
| --- | --- | --- | --- | --- | --- | --- | --- | --- | --- | --- | --- |
| 1 | LOS | hypotension | Serratia | 1 | UVC | 188 | 36 | 23 | 7.33 | 1.8 | 4.9 |
| 2 | LOS | hypotension | Serratia | 1 | cannula | 187 | 35 | 22 | 7.15 | 4.2 | 7.9 |
| 3 | LOS | hypotension | Klebsiella | 1 | cannula | 174 | 29 | 17 | 7.12 | 6.9 | 21.6 |
| 4 | LOS | hypotension | CONS | 1 | cannula | 141 | 37 | 13 | 7.22 | 1.0 | 5.4 |
| 5 | LOS | hypotension | CONS | 2 | PICC | 157 | 39 | 15 | 7.27 | 2.5 | 4.6 |
| 6 | LOS | hypotension | Serratia | 1 | cannula | 187 | 37 | 23 | 7.15 | 2.2 | 8.2 |
| 7 | LOS | hypotension | E. Coli | 1 | UVC | 161 | 46 | 24 | 7.20 | 3.3 | 3.6 |
| 8 | LOS | poor perfusion | Serratia | 2 | UVC | 151 | 48 | 33 | 7.28 | 1.4 | 3.7 |
| 9 | LOS | poor perfusion | E. Coli | 1 | cannula | 171 | 48 | 31 | 7.33 | 1.9 | 5.2 |
| 10 | LOS | poor perfusion | CONS | 1 | cannula | 181 | 50 | 18 | 7.17 | 3.9 | 4.5 |
| 11 | LOS | tachycardia | no growth | 1 | cannula | 175 | 77 | 41 | 7.31 | 1.6 | 5.0 |
| 12 | LOS | tachycardia | CONS | 1 | PICC | 190 | 65 | 33 | 7.35 | 1.7 | 3.5 |
|  |  |  |  |  |  |  |  |  |  |  |  |
| 13 | Gastroschisis | poor perfusion |  | 1 | cannula | 137 | 45 | 24 | 7.23 | 4.9 | 3.9 |
| 14 | Gastroschisis | poor perfusion |  | 1 | cannula | 129 | 45 | 29 | 7.23 | 1.4 | 3.0 |
| 15 | cong. CMV | poor perfusion |  | 1 | UVC | 135 | 51 | 23 | 7.41 | 2.1 | 7.7 |
| 16 | Meconium | tachycardia |  | 3 | UVC | 174 | 43 | 30 | 7.15 | 3.3 | 36.6 |
| 17 | Birth asphyxia | hypotension |  | 2 | UVC | 142 | 32 | 22 | 7.14 | 7.0 | 4.3 |

Table 2. (Supplemental material) Patient characteristics before the mini-fluid challenge. HR, heart rate; SBP, systolic blood pressure; DBP, diastolic blood pressure, OSI, oxygen saturation index derived from 2 x (mean airway pressure x FiO_2_ / pre-ductal saturation). LOS, late onset sepsis; CMV, cytomegalovirus; CONS, coagulase negative staphylococcus; UVC, umbilical venous line; PICC, peripheral inserted central catheter.
